# Epidemiology and associations with climatic conditions of *Mycoplasma pneumoniae* and *Chlamydophila pneumoniae* infections among Chinese children hospitalized with acute respiratory infections

**DOI:** 10.1186/1824-7288-39-34

**Published:** 2013-05-25

**Authors:** Zhengrong Chen, Wei Ji, Yuqing Wang, Yongdong Yan, Hong Zhu, Xuejun Shao, Jun Xu

**Affiliations:** 1 Department of Respiratory Disease, Children’s Hospital Affiliated to Soochow University, No. 303 JingDe Road, Suzhou, Jiangsu Province 215003, China; 2 Laboratory of Molecular Biology, Children’s Hospital Affiliated to Soochow University, Suzhou, China

**Keywords:** Acute respiratory tract infection, Mycoplasma pneumoniae, Chlamydophila pneumoniae, Epidemiology, China

## Abstract

**Background:**

The incidence of severe acute respiratory tract infections in children caused by *Mycoplasma pneumoniae* (syn. *Schizoplasma pneumoniae*) and *Chlamydophila pneumoniae* (*formerly Chlamydia pneumoniae*) varies greatly from year to year and place to place around the world. This study investigated the epidemiology of *M*. *pneumoniae* and *C*. *pneumoniae* infections among children hospitalized with acute respiratory infections in Suzhou, China in the year 2006, and associations between incidence rates and climatic conditions.

**Methods:**

Nasopharyngeal aspirates obtained from 1598 patients (aged 26.4 ± 28.3 months; range, 1 month to 13 years) were analyzed with real-time PCR and ELISA. Meteorological data were obtained from the weather bureau.

**Results:**

About 18.5% of patients were infected with *M*. *pneumoniae* and, *C*. *pneumoniae*, or both. Isolated *M*. *pneumoniae* infection was positively correlated with increasing age (χ^2^ = 34.76, *P* < 0.0001). Incidence of *M*. *pneumoniae* infection was seasonal with a peak in summer (*P* < 0.0001) and minimum in winter (*P* = 0.0001), whereas *C*. *pneumoniae* infection was low only in autumn (*P* = 0.02). Monthly mean temperature was strongly correlated with the incidence of *M*. *pneumoniae* infection (*r* = 0.825, *P* = 0.001).

**Conclusions:**

*M*. *pneumoniae* and *C*. *pneumoniae* are important infectious agents in hospitalized children with acute respiratory tract infections. *M*. *pneumoniae* infection showed a strong direct correlation with environmental temperature.

## Introduction

The atypical pathogens *Mycoplasma pneumoniae* (syn. *Schizoplasma pneumoniae*), *Chlamydophila pneumoniae* (*Chlamydia pneumoniae*), and *Legionella pneumophila* cause mild, moderate, or severe acute respiratory tract infections (ARTIs) in children, although *Legionella pneumophila* infection is more common in adults. These infections occur worldwide [1-3]. Cyclical outbreaks of *M*. *pneumoniae* infections can be expected on average every 3–7 years, but at any given time may account for as many as 40% of community-acquired pneumonia cases [3]. The prevalence of *C*. *pneumoniae* in children with ARTIs varies from 0 to 44% [4].

Studies of possible associations between the epidemiology of atypical pathogens and meteorological conditions (e.g., temperature, humidity, rainfall, amount of solar radiation and wind velocity) are few. However, it was recently reported that the community incidence of pneumonia due to *M*. *pneumoniae* increased weekly by 16.9% for every 1°C increase in the average temperature, and by 4.1% for every 1% increase in relative humidity [5]. Community-acquired *M*. *pneumoniae* or *C*. *pneumoniae* infections affect mainly preschool- and school-aged children and young adults. Few studies have reported the frequency of *M*. *pneumoniae* and *C*. *pneumoniae* infections in infants [6].

Clinically it is difficult to distinguish *M*. *pneumoniae* from *C*. *pneumoniae* infections and hence laboratory tests are essential in identifying these pathogens. Serological detections, although commonly used, are complicated by false negative results in the early acute phase of infection, and the difficulty in obtaining convalescent serum during hospital stays of one week or less. Polymerase chain reaction (PCR) is suitable for rapid diagnosis of these infections, even when the colonization rate is only 1-2% of the population [7-9]. Combining both PCR and serology seems to be a more reliable diagnostic approach.

The incidence rate of childhood ARTIs due to these pathogens is very different from one country to another [2-6]. Our purpose was to use PCR to identify and determine the percentage of ARTIs that were due to *M*. *pneumoniae* and *C*. *pneumoniae*, especially in infants, and investigate the relatedness between their epidemiology and meteorological conditions in Suzhou, Jiangsu Province, China. In addition we compared the clinical characteristics of *M*. *pneumoniae* and *C*. *pneumoniae* infections, host immune state, radiographic appearance, and tidal breathing measurements.

## Materials and methods

### Study population

From 1 January 2006 to 31 December 2006, 1598 consecutive children with ARTIs admitted to Children’s Hospital affiliated with Soochow University were enrolled and evaluated prospectively. These children were hospitalized because of prolonged fever (>3 d), severe symptoms of cough, wheeze, tachypnea, and chest retractions. The clinical outcome and diagnosis for all children were obtained after discharge from the hospital. The discharge diagnosis was based on standard clinical criteria made by attending physicians. Upper respiratory tract infection was diagnosed if a child had nasal obstruction, nasal discharge, fever, or sore throat. Lower respiratory tract infection was diagnosed when wheeze, tachypnea, chest retractions, abnormal auscultatory findings, and radiologic evidence of a lower respiratory tract infection were present. Children were excluded from the study if they had proven chronic lung disease, immunodeficiency, congenital heart disease, or bronchopulmonary dysplasia. The Institutional Review Boards of Suzhou University approved the study protocol, and the parents or legal guardians of each child gave informed written consent.

## Methods

### Sample collection

Nasopharyngeal aspirate (NPA) samples were obtained from each patient within 24 hours of admission using a sterile plastic catheter introduced into the lower part of the pharynx via the nasal cavity. The samples were immediately transported to the Laboratory of Molecular Biology of our hospital for detection of *M*. *pneumoniae* and *C*. *pneumoniae* using PCR. Seven common respiratory virus (respiratory syncytial virus, influenza A and B, parainfluenza 1, 2, 3 and adenovirus ) using direct immunofluorescence and human metapneumovirus using RT-PCR described previously [10]. Blood samples were also obtained at admission and immediately sent to the Department of Biochemical Laboratory for routine blood, C-reactive protein, humoral and cell immunity, and alanine transaminase tests.

### DNA extraction

Each NPA sample was diluted in 2 mL of normal saline before centrifugation at 500 × *g* for 10 minutes. The resultant cell pellet was resuspended and then centrifuged at 12 000 × *g* for 5 minutes, followed by extraction of DNA from a 400-μL sample using DNA-EZ Reagents (Sangon Biotech, USA) in accordance with the manufacturer’s instructions. A final 200 μL of DNA was eluted and divided into 2 aliquots for PCR and stored at −20°C.

### Detection of M. *pneumoniae* gene by real-time PCR

Real-time PCR was performed to identify the P1 adhesion protein gene of *M*. *pneumoniae*, as described previously [11]. The forward and reverse primers were 5’-CCA ACC AAA CAA CAA CGT TCA-3’ and 5’-ACC TTG ACT GGA GGC CGT TA-3’, respectively, and the probe sequence was 5’-TCA ACT CGA ATA ACG GTG ACT TCT TAC CAC TG-3’. The fluorescent reporter dye at the 5’ end was 6-carboxyfluorescein (FAM) and the quencher at the 3’ end was 6-carboxytetramethylrhodamine (TAMRA). The PCR reactions consisted of a 21-μL PCR mast mixture (Shenyou Biotechnology, Shanghai, China) including primers and probes combined with 3 μL of the sample DNA and 1 U GoTaq DNA Polymerase (Promgea, Wisconsin, USA). Real-time PCR was performed using an iCycler iQ5 real-time PCR detection system (Bio-Rad, Hercules, CA, USA) and cycling conditions were: 2 min at 37°C; 10 min at 94°C, and 40 cycles of 10 s at 94°C, 30 s at 55°C, and 40 s at 72°C.

Quantification curves were plotted using several concentrations of control plasmids containing the target gene.

### Detection of C. *pneumoniae* gene by nested-PCR

A different aliquot of extracted DNA from the NPA sample was used for detecting the gene of the major outer membrane protein of *C*. *pneumoniae* by nested touchdown PCR, as described previously [12]. The external and internal primers were: Cpex-F 5’-TTA CAA GCC TTG CCT GTA GG-3’; Cpex-R 5’-GCG ATC CCA AAT GTT TAA GGC-3’; Cpin-F 5’-TTA TTA ATT GAT GGT ACA AT A-3’; and Cpin-R 5’ATC TAC GGC AGT AGT ATA GTT-3’. Amplifications were performed in a thermal cycler (GeneAmp PCR System 9600, Applied Biosystems). For the first amplification, 0.4 μM of each CPex-F and Cpex-R primer and 2 U GoTaq DNA polymerase (Promgea, Madison, WI, USA) were included in these touchdown PCR reactions. The annealing temperature was lowered 1°C every 2 cycles, from 65°C until touching down at 55°C, at which temperature 20 more cycles were performed. The denaturation and extension temperature were constant at 94°C and 72°C, respectively. One microliter of the products of the first round of amplification by external primers were added into the PCR reactions of the second round amplification using internal primers. The second PCR cycling conditions were: 2 min at 95°C; and 30 cycles of 1 min at 94°C, 1 min at 50°C, and 1 min at 72°C. The nested PCR products were separated via 1.5% agarose gel electrophoresis and visualized using ethidium bromide staining.

### Serology testing for M. *pneumoniae* and C. *pneumoniae*

The presence of specific IgM and IgG antibodies against *M*. *pneumoniae* were investigated in serum samples of patients using a commercial ELISA kit (Serion ELISA classic *M*. *pneumoniae* IgG/IgM, Institute Virion\Serion, Germany). IgA and IgG antibodies against *C*. *pneumoniae* were detected with Serion ELISA classic *C*. *pneumoniae* IgA/IgG Kits. Evidence of acute *M*. *pneumoniae* infection was defined as either a single positive serum IgM (cutoff 13 U/mL) or a 4-fold increase in IgG in convalescent serum obtained a week after admission. Acute *C*. *pneumoniae* infection was defined as either a single positive serum IgA (cutoff 3 U/mL) or a 4-fold increase in IgG in convalescent serum.

### Diagnostic criteria for M. *pneumoniae* and C. *pneumoniae* acute infection

Acute infection due to *M*. *pneumoniae* or *C*. *pneumoniae* was confirmed when any of the following was determined: NPA samples proved positive via PCR, serum samples positive for IgM or IgA, respectively, or IgG increasing 4-fold from the acute to the convalescent phases.

### Tidal breathing measurements

Measurements of tidal breathing in most infants were performed during natural and quiet sleep as assessed by behavioral criteria [13]. Tidal breathing flow-volume loops were obtained and analyzed with a commercially available pediatric pulmonary function device (ECO Medics, V’max 26, Switzerland). Tidal breathing measurements included flow and volume signals, and were assessed by professional staff. Lung function was graded as normal, mild, moderate, severe, or extremely severe according to three-component score system which could reflect underlying obstructive airways disease [14,15] as shown in Table 1.

**Table 1:** **Three**-**component score system for assessing tidal lung function**

**Parameters**	**Scores**			
	**0**	**1**	**2**	**3**
PTEF/t-PTEF(mL/s^2^)	<3	3-6	7-10	>10
25TEF/PTEF (%)	>80	60-80	40-60	<40
t-PTEF/t-E (%)	>30	20-30	10-20	<10

The score for 5 levels of tidal lung function. Normal: 0, Mild: <3, moderate: 3–5, Severe: 6–7, Extremely severe: 8–9. *PTEF *peak tidal expiratory flow, *t*-*PTEF* time to peak tidal expiratory flow, *25TEF* tidal expiratory flow at 25% of tidal volume remaining, *t*-*E*, expiratory time.

### Meteorological data

**Figure 1:**
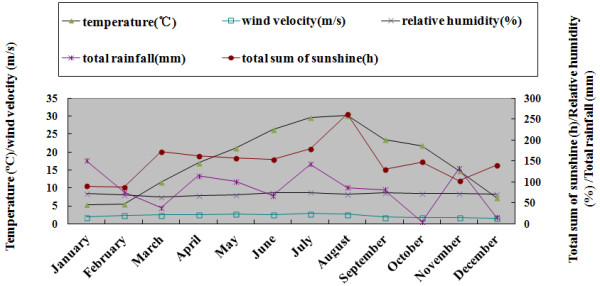
**The monthly data for meteorological variables in Suzhou, China.**

The primary investigator obtained data regarding monthly mean temperatures, relative humidity, rainfall, solar radiation, and mean wind velocity from the local weather bureau for lat 31.19° N, long 120.37° E. Suzhou has a subtropical climate and the monthly data for meteorological variables were shown in Figure 1.

### Statistical analyses

Categorical data were analyzed using the Cochran-Mantel-Haenszel statistic or the chi-squared (χ^2^) or Fisher’s exact tests. The continuous variables were compared using analysis of variance (ANOVA). The Kruskal-Wallace test was used if the data were abnormal in distribution or nonparametric. The associations between meteorological conditions and the prevalence of the 2 pathogens were evaluated using Spearman’s rank correlations. A *P*-value < 0.05 was considered statistically significant. All analyses were performed using the Statistical Package for SAS for windows, version 8.2 (SAS, USA).

## Results

### Epidemiology

Of the 1598 children admitted to our hospital for ARTIs, 957 (59.9%) were boys, and the ratio of boys to girls was 1.5:1. The mean age was 26.4 ± 28.3 months (range: 1 month to 13 years). Of all the children, 817 (51.1%) were aged < one year, 616 (38.5%) were 1–5 years, and 165 (10.3%) were >5 years old.

Twenty-three cases of *M*. *pneumoniae* infection and 6 cases of *C*. *pneumoniae* infection had co-infections with other viruses (data not shown), and cases of *M*. *pneumoniae* or *C*. *pneumoniae* coinfection with viruses were excluded in this study. ARTIs were caused by *M*. *pneumoniae* or *C*. *pneumoniae* in 295 (18.5%) of the patients. The mean age of these children was 33.1 ± 36.3 months. There were 171 boys, and the ratio of boys to girls was 1.4:1. Of the 295 infected with either or both of these pathogens, 199 (12.5%) were due to *M*. *pneumoniae* alone (mean age 35.8 ± 37.3 months), 81 (5.1%) were due to *C*. *pneumoniae* alone (mean age 27.3 ± 34.5 months), and 15 (5.1%) were co-infected (mean age 28.0 ± 29.7 months). The infection rate due to *M*. *pneumoniae* only was significantly and positively associated with increasing age (χ^2^ = 34.76, *P* < 0.0001). With regard to demographic data, no significant difference in gender or prematurity was found among the 3 etiological groups (Table 2).

**Table 2:** **The prevalence and demographic data of children hospitalized with *M***. ***pneumoniae *and *C***. ***pneumoniae *infection**

	***M. pneumoniae ***^**a**^	***C. pneumoniae ***^**b**^	**Co-infection **^**c**^
Age	n/total (%)	n/total (%)	n/total (%)
<1 year	80/817 (9.8)	40/817 (4.9)	8/817 (0.1)
1 to 5 years	75/616 (12.2)	30/616 (4.9)	5/616 (0.1)
>5 years	44/165 (26.7)	11/165 (6.7)	2/165 (0.1)
Total	199/1598 (12.5)	81/1598 (5.1)	15/1598 (0.1)
Demographics	n (%)	n (%)	n (%)
Total (male)	116 (58.3)	46 (56.8)	9 (60)
Prematurity	12 (6.0)	4 (4.9)	1 (6.7)
PICU Admission	1 (0.5)	0 (0)	0 (0)

^a^ χ^2^_CMH_ = 35.90, *P* < 0.0001; ^b^ χ^2^_CMH_ = 0.98, *P* = 0.614; ^c^ χ^2^_CMH_ = 0.25, *P* = 0.881 compared among three age groups (Cochran-Mantel-Haenszel Statistic test). Data are presented as number (percentage) or mean ± SD.

### Seasonality and correlations with meteorological conditions

**Figure 2:**
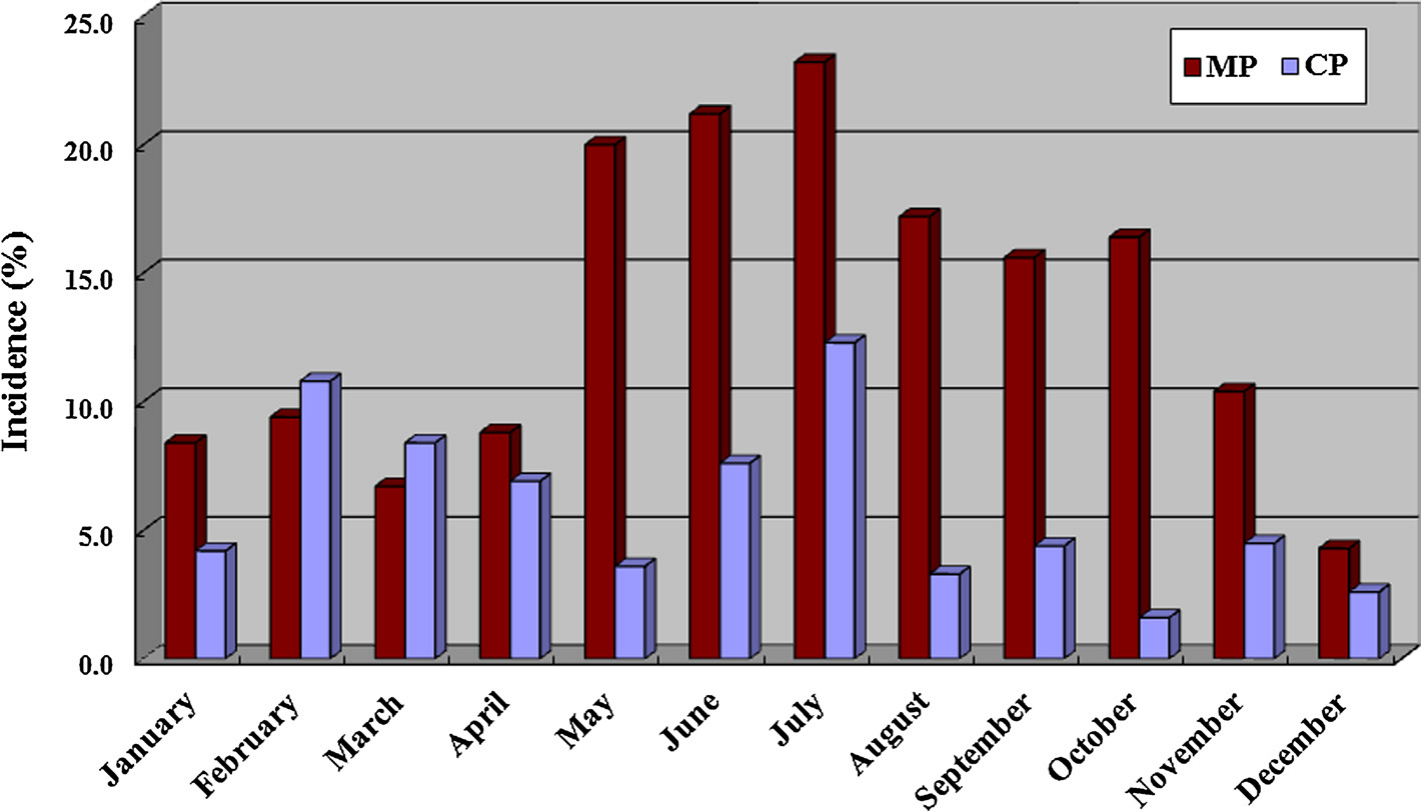
**The seasonality of *M. pneumoniae *and *C. pneumoniae *infections in hospitalized children.** MP, *M. pneumoniae*; CP, *C. pneumoniae*.

Both *M*. *pneumoniae* and *C*. *pneumoniae* infections occurred throughout the year. The *M*. *pneumoniae* infection rate reached a maximum in summer (*P* < 0.0001) and a minimum in winter (*P* = 0.0001), whereas the *C*. *pneumoniae* infection rate was lowest in autumn (*P* = 0.02; Table 3). The peak number of *M*. *pneumoniae* infections occurred in July (23.2% or 32/138) and were the lowest (4.3% or 5/117) in December. *C*. *pneumoniae* infections were also high in July (12.3% or 17/138; Figure 2).

**Table 3:** **The seasonality and correlations with climatic conditions of *M***. ***pneumoniae *and *C***. ***pneumoniae *infection in hospitalized children**

	*** M. pneumoniae***		*** C. pneumoniae***	
Seasons	n (%)		n (%)	
Spring (Mar-May)	43 (11.0)		26 (6.6)	
Summer (Jun-Aug)	86 (20.4) ^a^		32 (7.6)	
Autumn (Sep-Nov)	57 (13.9)		15 (3.6) ^c^	
Winter (Dec-Feb)	28 (7.5) ^b^		23 (6.1)	
Meteorological conditions	* r*	* P*	* r*	* P*
Temperature (°C)	0.825	0.001	−0.021	0.948
Relative humidity (%)	0.527	0.782	0	1.0
Rainfall (mm)	0.189	0.557	0.294	0.354
Sum of sunshine (h)	0.392	0.208	0.070	0.829
Wind velocity (m/s)	0.629	0.028	0.443	0.149

^a^*P* < 0.0001; ^b^*P* = 0.0001; ^c^*P* = 0.02 (prevalence of infections in a particular season compared to the mean prevalence in all other seasons). *r* Spearman coefficient.

Rates of *M*. *pneumoniae* infection in hospitalized children in Suzhou correlated strongly with monthly mean temperature, and weakly with monthly mean wind velocity (Table 3). There was no significant correlation between meteorological conditions and *C*. *pneumoniae* infections.

### Clinical manifestations

There was no significant difference in terms of clinical manifestations among the groups except for fever (χ^2^ = 7.824, *P* = 0.02; Table 4). Children co-infected with *M*. *pneumoniae* and *C*. *pneumoniae* were found to have fever more frequently. There were no significant differences in length of hospital stay among the different groups. All patients were cured or improved.

**Table 4:** **Clinical manifestations of children hospitalized with *M***. ***pneumoniae *and *C***. ***pneumoniae *infection**

	***M. pneumoniae***	***C. pneumoniae***	**Co-infection**
Hospitalization (d)	7.5 ± 2.3	7.3 ± 1.9	8.1 ± 2.3
Clinical manifestations, n (%)			
Cough	189 (95.0)	75 (92.6)	14 (93.3)
Wheezing	57 (28.6)	31 (38.3)	6 (40)
Nose running	40 (20.1)	21 (25.9)	5 (33.3)
Fever	114 (57.3) ^a^	36 (44.4)	12 (80) ^b^
Dyspnea	12 (6.0)	4 (4.9)	1 (6.7)
Anorexia	22 (11.1)	7 (8.6)	3 (20)
Gastrointestinal	30 (15.1)	17 (21.0)	4 (26.7)
Tachypnea	31 (15.6)	12 (14.8)	3 (20)
Cyanosis	2 (1.0)	0 (0)	0 (0)
Other diagnoses, n (%)			
URTI	10 (5.0)	8 (9.9)	1 (6.7)
LRTI	171 (85.9)	66 (81.5)	13 (86.7)
Asthma exacerbation	18 (9.0)	7 (8.6)	1 (6.7)

^a^*M*. *pneumoniae* group compared with *C*. *pneumoniae* group, *P* = 0.05.

^b^ Co-infection group compared with *C*. *pneumoniae* group, *P* = 0.011.

*URTI*, upper respiratory tract infection; *LRTI*, lower respiratory tract infection.

### Laboratory findings

There were no significant differences in laboratory findings (routine blood, C-reactive protein level, humoral and cell immunity, alanine transaminase, radiographic data, and tidal lung function test) among the 3 groups (Table 5).

**Table 5:** **No significant differences in laboratory findings of children hospitalized with *M***. ***pneumonia *and *C***. ***pneumoniae *infection**

	***M*. *pneumoniae***	***C*. *pneumoniae***	**Co-infection**
	**(n = 199)**	**(n = 81)**	**(n = 15)**
Cell immunity			
CD3+ (%)	63.6 ± 9.4	65.3 ± 9.3	61.9 ± 8.3
CD3 + CD4+ (%)	36.8 ± 8.2	37.5 ± 8.6	33.9 ± 4.4
CD3 + CD8+ (%)	22.9 ± 6.7	24.1 ± 5.7	23.6 ± 8.1
CD4/CD8 (%)	1.8 ± 0.8	1.7 ± 0.7	1.6 ± 0.6
CD3-CD19+ (%)	26.2 ± 9.8	26.1 ± 8.9	29.7 ± 9.2
CD3-CD (16 + 56+) (%)	8.8 ± 5.7	6.6 ± 3.9	6.8 ± 2.8
CD19 + CD23+ (%)	6.6 ± 3.6	6.9 ± 2.9	7.8 ± 2.5
CD4 + CD25+ (%)	7.4 ± 3.5	6.0 ± 3.7	7.0 ± 2.4
Chest X-rays (LRTI) *	n = 171	n = 66	n = 13
Lobar consolidation (%)	23 (13.5)	7 (10.6)	2 (15.4)
Infiltration/opacities (%)	105 (61.4)	41 (62.1)	8 (61.5)
Hyper aeration (%)	16 (9.4)	10 (15.2)	2 (15.4)
Interstitial lesions (%)	59 (34.5)	25 (37.9)	4 (30.8)
Pleural reaction (%)	4 (2.3)	0 (0)	1 (7.7)
Tidal lung function (LRTI)	n = 82	n = 54	n = 9
Normal (0)	13 (15.9)	9 (16.7)	1 (11.1)
Mild (1–3)	22 (26.8)	6 (11.1)	1 (11.1)
Moderate (4–5)	23 (28.0)	18 (33.3)	1 (11.1)
Severe (6–7)	20 (24.3)	17 (31.5)	4 (44.4)
Extremely severe (8–9)	4 (4.9)	4 (7.4)	2 (22.2)

*Chest X-rays and tidal lung function were only performed on children with *LRTI*. *CD* cluster of differentiation, *LRTI* lower respiratory tract infection.

## Discussion

*M*. *pneumoniae* and *C*. *pneumoniae* are two common atypical pathogens that cause ARTIs in children worldwide. Infections may be endemic or epidemic. In the present study, the total rate of *M*. *pneumoniae* and *C*. *pneumoniae* infections in hospitalized children with ARTIs in Suzhou, China for the year 2006 was 18.4% (295/1598), in which 12.5% and 5.1% were caused only by *M*. *pneumoniae* or *C*. *pneumoniae*, respectively. In other recent studies in which real-time PCR was used for identification, Defilippi et al. [16] in Genoa, Italy reported an infection rate for *M*. *pneumoniae* in children of 11.8% and in Tokyo, Japan Hamano-Hasegawa et al. [17] found a rate of 14.8%. The rates of *M*. *pneumoniae* infection found via real-time PCR tests in Suzhou, China in the years 2007 and 2008 were 5.39% and 6.36%, respectively (unpublished results). Hosker et al. [18] reported an epidemic of *M*. *pneumoniae* infection in Hong Kong with a rate of 15% to 20% that occurred from 1986 to 1988, which was similar to our study.

Apparent seasonality of *M*. *pneumoniae* infection was observed in this study, with a peak level in summer and the highest rate in July. When the disease is endemic, seasonality may not be a factor, but when it is an epidemic more cases occur in the summer or early autumn [3,16]. We observed a similar trend in this study. Onozuka et al. [5] found a strong correlation between temperature and relative humidity and the number of *M*. *pneumoniae* pneumonia cases in hospitalized children younger than 15 years in Japan, while another study in Germany did not show any correlation [19]. However, we presume that climatic conditions in our area are similar to Japan, and different from the northwestern region of Germany where the range of temperatures across the seasons is not as great.

Regarding *C*. *pneumoniae* infections, the 6.0% rate in children with ARTIs found in the present study is similar to the 6.7% (4/112) found in a study performed by Kurz et al. [20] in Vienna, Austria, and lower than the 9.3% reported by Schmidt et al. [21] for Greifswald, Germany. No seasonality or correlation with climatic conditions was found in our study. Studies conducted in various countries have shown that *M*. *pneumoniae* and *C*. *pneumoniae* infections are most common in school-aged children, followed by children from 1 to 5 years old, but rare in infants [16,21-24]. In contrast, we found that *M*. *pneumoniae* (10.8%) and *C*. *pneumoniae* (5.9%) infections are also common in infants. The high population density in China, which increases exposure and the chance of infection, may be an important influence in acquiring these infections.

The use of PCR in the present study provided rapid and specific diagnosis of *M*. *pneumoniae* and *C*. *pneumoniae* infections. A recent retrospective study comparing the diagnostic value of PCR to the indirect particle agglutination antibody test for *M*. *pneumoniae* infection in children from NPA samples found that PCR provided a more rapid diagnosis, particularly in young children (*P* = 0.003), immunocompromised patients (*P* = 0.019), and during the early stage of the disease [25]. When PCR is combined with serological tests the yield increases further [26].

In the present study, the clinical manifestations and laboratory findings did not differ between *M*. *pneumoniae* and *C*. *pneumoniae*. Cough and fever were the most common symptoms. Fever was noticed more frequently in children with co-infection, although co-infection did not increase the severity of clinical manifestations. The rate of wheezing in *M*. *pneumoniae* or *C*. *pneumoniae* infections was higher than that reported by Esposito et al. [27], and the rates of dyspnea and tachypnea were lower. The reason for this may be that the present study included upper respiratory infection, bronchitis, and asthma exacerbation within the definition of ARTIs.

The 8.8% (26/295) incidence of *M*. *pneumoniae* or *C*. *pneumoniae* infections with asthma exacerbation in this study indicates a possible role of infection by these atypical pathogens in asthma exacerbation. Recently, it was reported that in asthma *M*. *pneumoniae* and *C*. *pneumoniae* appeared to be involved more with asthma persistence than exacerbation [28].

In previous studies, no significant difference was found between *M*. *pneumoniae* and *C*. *pneumoniae* infections with regard to white blood cell count, C-reactive protein, percentage of neutrophils, platelets, or radiographic data [16,27,29]. To the best of our knowledge, this study is the first to analyze the humoral and cell immunity status and tidal lung function of children with *M*. *pneumoniae* and *C*. *pneumoniae* infections. Although no significant difference in immunity status existed between the patients infected with the two pathogens, some studies showed a depressed humoral and cellular immunity in children with *M*. *pneumoniae* infection compared to healthy children [30,31]. Because of the difficulty in distinguishing *M*. *pneumoniae* infection from *C*. *pneumoniae* on the basis of clinical manifestations or laboratory findings, PCR tests are very useful [32].

The limitations of our study include a probable bias, as the study was conducted only in hospitalized children and not outpatients. In addition, a study period of only one year is relatively short to assess the epidemiology of *M*. *pneumoniae* and *C*. *pneumoniae* infection.

## Conclusion

*M*. *pneumoniae* infections in children demonstrated a definite seasonality, a strong correlation with the temperature, and a weak correlation with wind velocity. Consistent with former studies, ours indicates that *M*. *pneumoniae* and *C*. *pneumoniae* are important in children with ARTIs who are younger than 5 years old, and especially in infants. Clinical manifestations and laboratory findings were informative, but real-time PCR and nested-PCR provide adequate rapid and specific diagnosis of *M*. *pneumoniae* and *C*. *pneumoniae* infections.

## Competing interests

The authors declare that they have no competing interests.

## Authors’ contributions

ZC: carried out the molecular genetic studies, participated in the sequence alignment and drafted the manuscript. WJ: design of experiment and drafted the manuscript. YW: drafted the manuscript. YY: design of experiment and drafted the manuscript. HZ: carried out the molecular genetic studies. XS: carried out the molecular genetic studies, participated in the sequence alignment. JX: carried out the molecular genetic studies, participated in the sequence alignment. All authors read and approved the final manuscript.
